# Cefaclor as a first-line treatment for acute uncomplicated cystitis: a retrospective single-center study

**DOI:** 10.1186/s12894-020-00605-6

**Published:** 2020-04-06

**Authors:** Dai Koguchi, Yasukiyo Murakami, Masaomi Ikeda, Masato Dobashi, Junichiro Ishii

**Affiliations:** Department of Urology, International University of Health and Welfare Atami Hospital, 13-1 Higashikaiganchou Atami, Shizuoka, 413-0012 Japan

**Keywords:** Acute uncomplicated cystitis, Antibiotics, Cefaclor, Clinical efficacy

## Abstract

**Background:**

Wide-spectrum antibiotics have been favored to treat acute uncomplicated cystitis (AUC) for a long time, leading to the emergence of multi-drug resistant bacteria. We hypothesize that narrow-spectrum antibiotics might mitigate the issue and aim to investigate the clinical efficacy of cefaclor in patients with AUC.

**Methods:**

We retrospectively reviewed the clinical data of female outpatients with AUC treated with cefaclor and evaluated the safety and clinical efficacy. Clinical cure was defined as the elimination of clinical symptom under 4 white blood cells (WBCs) per high power field on microscopy.

**Results:**

Overall, 223 women with AUC were enrolled. *Escherichia coli* was the dominant pathogen (*n* = 160; 68.6%), followed by *Klebsiella* species and *E. coli*-extended spectrum β-lactamase (ESBL) (*n* = 19; 8.1% and *n* = 18; 7.7%). Overall success rate was 94.0% (*n* = 219) and susceptibility rate of cefazolin was 84.1%, which was close to that of levofloxacin (82.9%). Ampicillin showed the lowest rate of 63.7% with a significantly greater resistance rate of 35.3% among all antibiotics (*P* < 0.001). In the subgroup analysis, the success rate in patients with resistance to levofloxacin or cefazolin was 100% (*n* = 24) or 93.3% (*n* = 14). The rate in patients with resistance to both antibiotics was 60.0% (*n* = 9), and the pathogens in the other 40.0% (*n* = 6) of patients with treatment failure were *E. coli*-ESBL.

**Conclusion:**

Cefaclor showed excellent efficacy in AUC patients, even in those with in vitro resistance to cefazolin or levofloxacin. Cefaclor may be considered as a first-line option in patients with AUC and a second-line option for those with levofloxacin treatment failure.

## Background

Urinary tract infections (UTIs) are the most frequently reported infections, and acute uncomplicated cystitis (AUC) is the most common presentation of UTIs [1, 2]. Approximately 50% of women will experience at least one UTI during their lifetime, and 25% will have recurrent infections [3]. Given that successive or newer generations of antibiotics tend to have increased activity against Gram-negative bacilli, and wide-spectrum antibiotics have been favored for a long time, AUC drives antibiotic use around the world, leading to the increasing antimicrobial resistance of bacterial pathogens [4].

In Japan, fluoroquinolones have been widely introduced as the treatment for AUC, because levofloxacin (LVFX) was recommended as the first-line therapy for pre-menopausal women and as the second-line treatment for the menopausal women according to the Japanese Association for Infectious Diseases and Japanese Society of Chemotherapy guidelines 2015 (*JAID/JSC)* [5]. *However, f*luoroquinolone resistance among uropathogens such as Enterobacteriaceae is particularly problematic. Although the rates of the drug resistance among Enterobacteriaceae in the community had remained low at < 1% for decades, the rate has skyrocketed in recent years, ranging from 10 to 30% in developed countries [6]. Additionally, the American Urological Association is strengthening the current warnings that fluoroquinolone antibiotics should be avoided for the treatment of AUC due to significant decreases in blood sugar and certain mental health side effects [7].

There has also been an increasing issue on multi-drug resistant (MDR) bacteria. Typically, MDR bacteria are associated with UTl infections. However, some MDR bacteria have become quite prevalent causes of community-acquired infections, spreading into the community, and is associated with increased morbidity, mortality, and healthcare costs [8]. In other words, patients with AUC should be managed in terms of not only the treatment of current cystitis but also the prevention of such pathogens in a prospective view. For example, the third-generation cephalosporin, which covers a variety of gram-negative rods and has also been frequently prescribed to outpatients with AUC as empirical therapy, has been reported to contribute to the production of extended spectrum β-lactamase (ESBL) [9].

These tendencies thus call for a new look at the therapeutic options, which have a relatively narrower spectrum, and we believe that one of the suitable alternatives is cefaclor (CCL), whose spectrum of activity is identical to that of cephalexin classified as the first-generation cephalosporins except that CCL also showed the added activity against β-lactamase-producing *Haemophilus influenzae* [10, 11]. Therefore, we aimed to evaluate the clinical efficacy of CCL in patients with AUC.

## Methods

This study was approved by the institutional review board of the International University of Health and Welfare Atami Hospital and was conducted in accordance with guidelines stipulated in the Declaration of Helsinki. In view of the retrospective nature of the study, obtaining patients’ informed consent was waived.

We retrospectively reviewed the clinical data of female outpatients with AUC, who were treated with CCL at the International University of Health and Welfare Atami Hospital between April 1, 2016 and March 25, 2019. Female patients aged 16 years or older were recruited for this study if they had the following conditions: i) symptoms of acute uncomplicated cystitis, such as dysuria, urinary urgency, and suprapubic pain occurring within 7 days before their visit to our hospital; ii) pyuria confirmed by reagent strip ≥5 white blood cells (WBCs) per high-power field (hpf) on microscopy of urine sediment, and iii) bacteriuria ≥10^4^ colony-forming units (cfu)/ml. We excluded the following patients: those who were pregnant, febrile, with urinary tract abnormalities including stones and hydronephrosis, multiple pathogens in a urine culture, diabetes mellitus, a malignancy under treatment, cerebrovascular disease, a history of antibiotic therapy within 4 weeks prior to visit to our hospital, malfunction of the heart, liver, or kidney, and on immunosuppressant therapy.

At the first visit, the urine specimens were subject to urinalysis and urine culture. Then, patients received oral CCL 750 mg per day, which is the standard dose in the Japanese health insurance system, for five to 7 days. They were asked to return to the hospital five to 7 days after the treatment for the assessment of drug safety and clinical efficacy by evaluating their urinalysis results and symptoms. Clinical cure was defined as the elimination of clinical symptom including dysuria, urinary urgency, suprapubic pain, and pyuria under 4 WBCs per hpf on microscopy. The susceptibility of each isolated pathogen to 6 antibiotics including ampicillin (ABPC), cefazolin (CEZ), levofloxacin (LVFX), *sulfamethoxazole*/*trimethoprim (ST), amikacin (AMK), and fosfomycin (FOM) was determined by using the MicroScan WalkAway 96 SI (Siemens, Munich, Bayern, Germany),* according to the Clinical and Laboratory Standards Institute (CLSI) Document M100-S22.

Statistical significance was determined using the chi-square test (or Fisher’s exact test, if appropriate) for categorical variables. All statistical analyses were performed with Stata version 13 for Windows (Stata, Chicago, IL, USA). All *P* values were two-sided, and *P* < 0.05 was considered statistically significant.

## Results

One patient was excluded from the present study due to nausea, which emerged on the day when CCL was prescribed, and an alternative antibiotic was introduced. Overall, 223 female patients with AUC were enrolled in this study, including 34 (14.6%) patients aged < 50 years and 199 (85.4%) aged > 50 years. Among the pathogens detected in the patients, as shown in Table 1, *Escherichia coli* was the dominant pathogen (*n* = 160; 68.6%) followed by *Klebsiella* species and *E. coli*-ESBL (*n* = 19; 8.1% and *n* = 18; 7.7%). The same distribution pattern of pathogens was also shown in patients aged > 50 years, and *E. coli* accounted for 65.8% (*n* = 131), *Klebsiella* species for 9.1% (*n* = 18), and *E. coli*-ESBL for 8.5% (*n* = 17) of cases. Significantly greater proportion of *E. coli* was found in patients aged < 50 years than in those aged > 50 years (*P* < 0.001), whereas each of the other five pathogens was found in each patient aged < 50 years.

**Table 1:** Pathogens found in this study

	All (*n* = 233)	< 50 years (*n* = 34)	> 50 years (*n* = 199)	*P* value
**Median age (IQR)**	75 (66–81)	37 (24–47)	77 (71–81)	
**Pathogen**				
*Escherichia coli*	160 (68.6)	29 (85.3)	131 (65.8)	0.027
*Klebsiella* species	19 (8.1)	1 (2.9)	18 (9.1)	0.33
*Escherichia coli*-ESBL	18 (7.7)	1 (2.9)	17 (8.5)	0.484
*Proteus* species	9 (3.8)	1 (2.9)	8 (4.0)	0.762
*Staphylococcus* species	6 (2.5)	1 (2.9)	5 (2.5)	0.884
*Streptococcus* species	3 (1.3)	1 (2.9)	2 (1.0)	0.752
*Citrobacter* species	5 (2.1)	0	5 (2.5)	N.A
*Enterococcus faecalis*	4 (1.7)	0	4 (2.0)	N.A
*Escherichia fergusonii*	3 (1.3)	0	3 (1.5)	N.A
*Corynebacterium*	2 (0.9)	0	2 (1.0	N.A
*Morganella morganii*	1 (0.5)	0	1 (0.5)	N.A
*Pseudomonas aeruginosa*	1 (0.5)	0	1 (0.5)	N.A
*Serratia marcescens*	1 (0.5)	0	1 (0.5)	N.A
*Shewanella algae*	1 (0.5)	0	1 (0.5)	N.A

Unless otherwise stated, values are medians with ranges in parentheses or number of patients with percentages in parentheses

*ESBL* Extended spectrum beta-lactamase, *N.A* Not applicable, *IQR* Interquartile range

The results of the antibiotic susceptibility test are presented in Table 2. Among all patients, the susceptibility rate of CEZ was 84.1%, which was close to that of LVFX (82.9%). ABPC showed the lowest rate of 63.7% (*P* = 0.027) with significantly greater proportion of the resistance rate of 35.3% among all antibiotics (*P* < 0.001), whereas ST, AMK, and FOM had > 90% (94.3, 99.1, 94.4%). The tendency of the greater resistance rate of ABPC for all pathogens was also applied to the analysis of *E. coli*, as shown in Table 3 (*P* < 0.001). In terms of age, there was no significant difference in the results of susceptibility test of each antibiotic in the analysis of the overall pathogens and *E. coli* between patients aged < 50 years and those aged > 50 years.

**Table 2:** Results of the antibiotic susceptibility test (%)

	All			< 50 years			> 50 years			
	S	I	R	S	I	R	S	I	R	*P* value
**CEZ**	84.1	2.1	13.8	86.2	6.2	6.2	83.6	1.6	14.8	0.269
**ABPC**	63.7	1.0	35.3	69.6	0	30.4	62.8	1.5	35.7	0.56
**LVFX**	82.6	0	17.4	88.2	0	11.8	82.1	0	17.9	0.616
**ST**	94.3	0	5.7	85.7	0	14.3	95.3	0	4.7	0.346
**AMK**	99.1	0	0.9	100	0	0	99.1	0	0.9	0.798
**FOM**	94.4	2.0	3.6	100	0	0	94.0	2.0	4.0	0.504

*S* Susceptible, *I* Intermediate, *R* Resistant, *ABPC* Ampicillin, *CEZ* Cefazolin, *LVFX* Levofloxacin, *ST Sulfamethoxazole/trimethoprim, AMK Amikacin, FOM Fosfomycin*

**Table 3:** Results of the antibiotic susceptibility test for *Escherichia* coli (%)

	All			< 50 years			> 50 years			
	S	I	R	S	I	R	S	I	R	*P* value
**CEZ**	95	2.5	2.4	89.3	7.1	3.6	96.4	1.4	2.2	0.268
**ABPC**	76.6	0.6	22.8	75.0	0	25.0	76.9	0.8	22.3	0.560
**LVFX**	87.4	12.6	0	89.3	0	10.7	87.0	0	13.0	0.616
**ST**	98	0	2	85.7	0	14.3	100	0	0	0.137
**AMK**	98.8	1.2	0	100	0	0	98.6	0	1.4	1
**FOM**	98.8	0	1.2	100	0	0	100	0	0	1

*S* Susceptible, *I* Intermediate, *R* Resistant, *ABPC* Ampicillin, *CEZ* Cefazolin, *LVFX* Levofloxacin, *ST Sulfamethoxazole/trimethoprim, AMK Amikacin, FOM Fosfomycin*

The success rate of CCL is shown in Table 4. Overall success rate was 94.0% (*n* = 219). Regarding gram staining, the cure rate of seven of the nine gram-negative pathogens and three of the five gram-positive pathogens was 100%. The remaining 6% (*n* = 14) had treatment failure with urinary symptoms at the follow-up examination. One (7.1%) of the 14 patients was aged < 50 years, and the causative pathogen was *E. coli*. The remaining 13 patients were aged > 50 years, and causative pathogens were *E. coli*-ESBL (*n* = 7: 50%), *E. coli* (*n* = 3: 21.4%), *Klebsiella pneumoniae* (*n* = 1: 7.1%), *Staphylococcus agalactiae* (*n* = 1: 7.1%), and *Enterobacter faecalis* (*n* = 1: 7.1%). None of the 14 patients with CCL treatment failure had pyelonephritis, and they were cured after receiving second-line antibiotic treatment; LVFX was introduced to *E. coli*-ESBL (*n* = 1), *E. coli* (*n* = 4), *Klebsiella pneumoniae* (*n* = 1), and *Staphylococcus agalactiae* (*n* = 1)*.* FOM was administered to *E. coli*-ESBL (*n* = 6) and ABPC to *Enterobacter faecalis* (*n* = 1: 7.1%).

**Table 4:** The success rate of cefaclor for all patients and that for patients with secondary treatment

	All (*n* = 233)		< 50 years (*n* = 34)		> 50 years (*n* = 199)		
	Success	Fail	Success	Fail	Success	Fail	*P* value
**All**	219 (94.0)	14 (6.0)	33 (97.1)	1 (2.9)	186 (93.5)	13 (6.5)	< 0.001
**Pathogens with the failure of cefaclor**							
*Escherichia coli*	156 (97.5)	4 (2.5)	33 (97.1)	1 (2.9)	128 (97.7)	3 (2.3)	0.975
*Klebsiella* species	18 (94.7)	1 (5.3)	1 (100)	0	17 (94.4)	1 (5.6)	0.971
*Escherichia coli*-ESBL	11 (61.1)	7 (38.9)	1 (100)	0	10 (58.8)	7 (41.2)	0.714
*Staphylococcus* species	5 (83.3)	1 (16.7)	2 (100)	0	3 (75.0)	1 (25.0)	0.741
*Enterococcus faecalis*	3 (75.0)	1 (25.0)	0	0	3 (75.0)	1 (25.0)	1

Unless otherwise stated, the number of patients with percentages in parentheses

*ESBL* Extended spectrum beta-lactamase

In the subgroup analysis of the efficiency of CCL in patients with resistance to specific antibiotics including LVFX and CEZ (Table 5), the success rate in patients with resistance to LVFX or CEZ was 100% (*n* = 24) or 93.3% (*n* = 14), respectively. Of these, AUC caused by *E. coli*-ESBL was cured in one patient with resistance to LVFX (100%; *n* = 1) and two in those with resistance to CEZ (66.6%; *n* = 2). The success rate in patients with resistance to both antibiotics was 60.0% (*n* = 9), and all pathogens in the other 40.0% (*n* = 6), who failed treatment were *E. coli*-ESBL.

**Table 5:** Efficiency of cefaclor in patients with in vitro resistance to specific antibiotics including levofloxacin and cefazolin

Antimicrobial agent	n	Success	Pathogen	n	Success
Levofloxacin	24 (10.3)	24 (100)	*Escherichia coli*	17 (70.8)	17 (100)
			*Escherichia coli*-ESBL	1 (4.1)	1 (100)
Cefazolin	15 (6.4)	14 (93.3)	*Escherichia coli*	3 (20.0)	3 (100)
			*Escherichia coli*-ESBL	3 (20.0)	2 (66.6)
Levofloxacin and cefazolin	15 (6.4)	9 (60.0)	*Escherichia coli*	1 (6.7)	1 (100)
			*Escherichia coli*-ESBL	14 (93.3)	8 (57.1)

Unless otherwise stated, number of patients with percentages in parentheses

*ESBL* Extended spectrum beta-lactamase

## Discussion

The present single-center study included a large cohort of 233 female patients with AUC and evaluated the clinical efficacy of CCL in the patients. Several results of the present study deserve attention. First, the present study showed the excellent efficacy of CCL in patients with AUC. The overall cure rate was 94%, and from the perspective of gram staining in the present study, each cure rate of gram-negative pathogens, except for *E. coli*-ESBL, showed approximately > 95%, and other five gram-positive pathogens also showed an acceptable efficacy rate, ranging from 75 to 100%. This is in line with the recent multi-institutional study including 155 Japanese patients with AUC [12]. With respect to the adverse events related to CCL, as previous studies described low incidence of side effects [2, 3], only one patient was excluded in this study due to nausea, which disappeared the next day after CCL discontinuation. Therefore, we believe that CCL is thus effective and tolerable in patients with AUC.

Second, CCL for AUC also had an effect even in patients with resistance to CEZ, as shown in the susceptibility test. In the current study, although 15 (6.3%) patients were revealed to be resistant to CEZ, 14 out of the 15 (93.3%) patients were treated by CCL, which may be explained by the striking pharmacokinetics of CCL [10, 11]. CCL was found to be rapidly absorbed in the gastrointestinal system; thus, its plasma concentration usually peaked within 1 hour after administration with no CCL detected in the serum at 6 hours. Then, the principal route of excretion of the drug was the urinary tract; thus, 60–80% of the drug could be found in the urine. Therefore, its urine concentration might be sufficient for treating most of the pathogens resistant to CEZ, as shown in the in vitro test; this bioavailability is higher than that of the third-generation cephalosporin [9]. Moreover, similarly, it is reasonable to speculate that this high bioavailability would contribute to the acceptable cure rate of 53.3% in patients with *E. coli*-ESBL in the current study. Although *E. coli*-ESBL is defined by the susceptibility test in vitro as resistant to almost all kinds of cephalosporin [13], there have been increasing evidence of positive impact of cephalosporin antibiotics on the pathogen, which supports our finding [14–16]. Cefmetazole (CMZ) in particular has been reported to be comparable with carbapenems as the definitive therapy for *E. coli*-ESBL in patients with nephritis and even those with bacteremia [15, 16]. However, the diversity of *E. coli*-ESBL including 300 strains in the world would lead to a variety of results in terms of the effect of CMZ, and no consensus has been reached until now [17].

Referring to the Japanese Association for Infectious Disease/Japanese Society of Chemotherapy (JAID/JSC) guidelines [5], CCL is recommended as the second-line treatment for premenopausal women with AUC based on two Japanese nationwide studies, which reported that approximately 10% of gram-positive cocci (GPC) were reported to be resistant to cephalosporins [18, 19]. However, the low prevalence of causative GPC in patients with AUC has been reported for decades. The Japanese nationwide studies reported it as 13.4%. A much lower rate was shown in the present study at 5.5%, consistent with the finding of a large randomized control trial reporting that GPC was found in 25 (3.8%) of the 661 female patients with AUC [20]. Therefore, as long as data on urine culture do not exist at the initial treatment of AUC, and resistance rate of cephalosporins for GPC was calculated to be approximately 10%, the introduction of wide-spectrum antibiotics under the assumption that such GPC resistant to narrower antibiotics may be unnecessary for almost all cases, or rather leading to a shortage of appropriate treatment options for serious infections [21]. Therefore, in the present study, the difference in the overall in vitro resistance rate of 13.8% and overall clinical success rate of 94.0% may be because of the high bioavailability of CCL; the gap between the results of the susceptibility test and clinical outcomes highlights the importance of pharmacokinetics and imperfection of susceptibility test in selecting the appropriate antibiotics [22]. Prospective studies are needed to investigate the association among susceptibility test, pharmacokinetics, and clinical outcomes.

For the last decade, the economical approach has also been highlighted in the choice of appropriate treatment for AUC because of rapidly increasing healthcare costs, which has been a significant issue worldwide [23]. The price of a 250-mg CCL capsule at the time of writing this article costs 48.0 Yen in Japan, whereas a 500-mg LVFX tablet costs 135.6 Yen; thus, one-week therapy of 750-mg CCL is 58.8 Yen more costly than that of LVFX. However, there is no doubt that the indiscriminate use of wide-spectrum antibiotics has been accepted as a definitive risk factor for the emergence of MDR pathogens [21], which have been reported to potentially worsen the severity of UTIs compared with pathogens without the resistance [24]. In other words, MDR would contribute to the high economic burden to the healthcare system due to frequent laboratory examinations, longer length of hospital stay, and administration of broad-spectrum antibiotics for worse infections [25]. A multinational observational study including 20 hospitals in eight countries showed that the presence of MDR was related to a higher cost [26]. In a report by the Canadian Committee on Antibiotic Resistance focusing on hospital-associated infections, especially methicillin resistant *Staphylococcus aureus*, antimicrobial resistance adds $8.7 to $13.9 million more indirect costs compared to infections without drug resistance [27]. In short, this economical concern supports the introduction of relatively narrow-spectrum antibiotics in patients with AUC in the long run. Additionally, duration of antibiotic treatment may also play an important role in both the economic status and the emergence of MDR pathogens [21]. In fact, studies showing the feasibility of a single three-gram dose of FOM in patients with AUC flourished recently for the reduction of economic burden and the prevention of MDR pathogens [28, 29]. To date, an interventional clinical trial evaluating short-term antibiotic treatment for urinary tract infection is ongoing (NCT03256825) [30].

Some limitations need to be considered when interpreting the results of our study. First, the study was retrospective in nature and relied on previously collected data. Second, although duration of CCL was not uniform in each case, all patients were treated with CCL for five to 7 days. Therefore, the slight difference of 2 days at most in the duration of the treatment would not significantly affect our result. Third, taking regional differences in antimicrobial resistance into consideration, the results of the present study may not be applicable to all institutions. However, as high bioavailability and narrower spectrum of CCL remain steady worldwide and for all time periods, CCL as the treatment of AUC is worthy of consideration in terms of the striking basic feature and prevention of MDR pathogens. Fourth, in spite of the lack of the data on CCL’s susceptibility due to the guideline of the CLSI Document M100-S22,

CEZ, which had as the same spectrum as CCL mainly except for *Haemophilus influenzae*, was tested in all patients. Hence, we believe that the current analysis using susceptibility test of CEZ is reliable to investigate the efficacy of CCL in patients with AUC.

## Conclusion

The present large single-center study showed the excellent efficacy and tolerance of CCL as the first-line therapy in patients with AUC. Notably, CCL for AUC was effective even in almost all patients with in vitro resistance to CEZ or LVFX, and found an acceptable success rate of > 50% even in those patients whose AUC was caused by *E. coli*-ESBL, which has been regarded as one of the most important public health threats. Therefore, CCL may be considered as a first-line treatment option in patients with AUC and as a second-line treatment option for those with LVFX treatment failure when the susceptibility test shows that the patient is resistant to LVFX, but susceptible to CEZ. Prospective *randomized studies* need to be carried out in order to draw definitive conclusions.

## Data Availability

The datasets used and/or analysed during the current study are available from the corresponding author on reasonable request.

## Abbreviations

ABPC: Ampicillin
*AMK*: *Amikacin*
AUC: Acute uncomplicated cystitis
CCL: Cefaclor
CEZ: Cefazolin
cfu: Colony-forming units
*CMZ*: Cefmetazole
CLSI: The Clinical and Laboratory Standards Institute
ESBL: *E. coli*-extended spectrum β-lactamase
*FOM*: *Fosfomycin*
GPC: Gram-positive cocci
hpf: High-power field
IDSA: The Infectious Diseases Society of America
*JAID/JSC*: The Japanese Association for Infectious Diseases and Japanese Society of Chemotherapy
LVFX: Levofloxacin
MDR: Multi-drug resistant
ST: S*ulfamethoxazole*/*trimethoprim*
UTI: Urinary tract infections
WBC: White blood cells
